# Development and validation of a multifactorial risk prediction model for breast cancer patients with co-occurring thyroid cancer: a retrospective matched case-control study

**DOI:** 10.3389/fonc.2026.1772910

**Published:** 2026-03-17

**Authors:** Junming Yin, Zhiwei Guo, Wen Yi, Ying He, Yi Luo, Kepeng Zhu, Songlin Yuan, Guocheng Du

**Affiliations:** 1 Department of Mastothyroid Vascular Surgery, Beijing Anzhen Nanchong Hospital of Capital Medical University & Nanchong Central Hospital, The Second Clinical Medical College of North Sichuan Medical College, Nanchong, Sichuan, China; 2 Department of Institute of Brain Function Rehabilitation and Imaging, Beijing Anzhen Nanchong Hospital of Capital Medical University & Nanchong Central Hospital, The Second Clinical Medical College of North Sichuan Medical College, Nanchong, Sichuan, China

**Keywords:** breast neoplasms, machine learning, risk prediction model, secondary primary neoplasms, thyroid neoplasms

## Abstract

**Objective:**

To develop and validate a multifactorial machine learning model predicting thyroid cancer (TC) co-occurrence risk in breast cancer (BC) patients.

**Methods:**

This single-center retrospective matched case-control study analyzed 400 BC patients (200 with co-occurring TC, 200 matched BC-only controls) diagnosed between 2012-2025. Predictors included demographic, clinical, hormonal, and tumor biological variables. After feature selection via LASSO regression to handle multicollinearity, four machine learning algorithms (logistic regression, random forest, XGBoost, SVM) were developed and optimized using Bayesian hyperparameter tuning with 5-fold cross-validation. Model performance was evaluated on a 30% independent test set using AUC-ROC, calibration curves, and decision curve analysis.

**Results:**

Multivariate analysis identified independent risk factors for TC co-occurrence: radiotherapy history (aOR = 3.42, 95% CI: 2.14–5.46), elevated TSH level (aOR = 2.01 per µIU/mL, 95% CI: 1.65–2.45), ER-positive status (aOR = 2.47, 95% CI: 1.43–4.28), family history of TC (aOR = 3.05, 95% CI: 1.55–6.00), and younger age at BC diagnosis (aOR = 1.07 per year decrease, 95% CI: 1.04–1.10). The XGBoost model demonstrated superior discrimination (test AUC = 0.874, 95% CI: 0.836–0.934) compared to other algorithms, with 86.7% accuracy, 83.3% sensitivity, and 90.0% specificity. Notably, subgroup analysis revealed enhanced predictive performance in patients with a history of radiotherapy (AUC = 0.921). Decision curve analysis confirmed clinical utility across threshold probabilities (20–80%), showing a superior net benefit for personalized risk stratification.

**Conclusion:**

The XGBoost-based model integrates radiotherapy exposure, hormonal profiles, and tumor biology to stratify TC risk in BC patients. It offers a clinically applicable tool for personalized surveillance, balancing early detection with resource optimization. External validation is warranted before implementation.

## 1 Introduction

Breast cancer (BC) and thyroid cancer (TC) are significant malignancies affecting women globally. In 2020, BC caused approximately 2.3 million new cases and 685,000 deaths, surpassing lung cancer as the most common female malignancy (1). BC represents 11.7% of all cancer diagnoses, while TC accounts for 3.0% (1). Improved cancer survival rates have revealed increased risk of second primary tumors, with BC survivors showing elevated TC incidence and TC survivors demonstrating higher BC occurrence (2, 3). This bidirectional association suggests shared pathogenic mechanisms requiring further investigation.

Current research implicates multifactorial mechanisms in BC-TC co-occurrence. Genetic susceptibility involves germline PARP4 mutations that predispose to both malignancies (4), while RET/PTC rearrangements, established drivers of thyroid cancer, function as estrogen-dependent kinases linked to poor prognosis in estrogen receptor-positive (ER-positive) BC (5–7). Dysregulation of the sodium/iodide symporter (NIS) appears in both lactating breast tissue and BC (8, 9), paralleling impaired iodide transport in papillary thyroid carcinoma (10), though its role in tumor angiogenesis remains debated (10). Estrogen signaling contributes significantly, where elevated endogenous or exogenous estrogen exposure promotes BC development (11), and functional estrogen receptors in TC modulate metastatic potential. Thyroid hormone receptors (TRs) demonstrate opposing roles, with thyroid hormone receptor beta (TRβ) suppressing tumor progression through PI3K/ERK pathway inhibition in both cancers (12), contrasting with thyroid hormone receptor alpha’s (TRα) oncogenic effects. Environmental factors, including ionizing radiation (13) and obesity (14), further increase the risk of co-occurrence.

Despite these mechanistic insights, clinical prediction tools for BC-TC co-occurrence remain underdeveloped. Previous studies, such as the population-based analysis by Zhang et al. (15), have primarily relied on conventional logistic or Cox regression methods to identify isolated risk factors. These approaches often overlook synergistic interactions between variables, such as the interplay between thyroid-stimulating hormone (TSH) elevation and ER status and fail to capture non-linear relationships essential for modeling complex comorbidities (16, 17). Consequently, predictive accuracy in high-dimensional clinical data remains limited.

To address these limitations, we developed the first multifactorial machine learning model integrating demographic, clinical, molecular subtype, and hormonal predictors of BC-TC co-occurrence. This study aims to identify independent risk factors through advanced modeling, validate a clinically applicable prediction tool, and elucidate key clinical associations to guide targeted surveillance strategies.

## 2 Methods

### 2.1 Study design

This single-center retrospective matched case-control study utilized anonymized electronic medical records (EMR) to identify and validate multifactorial risk predictors for BC patients developing co-occurring TC. Data were extracted from January 1, 2012, to March 31, 2025, at Beijing Anzhen Nanchong Hospital, Capital Medical University & Nanchong Central Hospital. The study protocol was approved by the Institutional Review Board of Beijing Anzhen Nanchong Hospital, Capital Medical University & Nanchong Central Hospital (Approval No: 2025-117), which granted a waiver of informed consent as the research involved only retrospective analysis of de-identified clinical data without any intervention, in accordance with local regulatory guidelines and the Declaration of Helsinki. All data were managed using encrypted databases with strict access controls to ensure patient confidentiality.

### 2.2 Study participants

#### 2.2.1 Patient identification and screening

Potential participants were identified through the institutional cancer registry database (2012–2025). EMR were systematically screened using ICD-10 codes for breast cancer (C50) and thyroid cancer (C73). A three-stage verification process was implemented: Initial algorithm-based screening for dual-coded cases (BC+TC); Manual chart review by two independent oncologists; Pathological confirmation via histopathology reports. To ensure the control group was strictly TC-free, we implemented a rigorous verification workflow. For the BC-only cohort, EMRs were reviewed for at least five years post-BC diagnosis (or until the study endpoint) to confirm the absence of thyroid-related ICD codes, suspicious neck imaging findings on ultrasound or CT reports, and pathology results. Any patient with suspicious thyroid nodules without definitive diagnostic exclusion during the follow-up period was excluded from the control group to minimize surveillance bias.

#### 2.2.2 Group stratification and control selection

**Figure 1:**
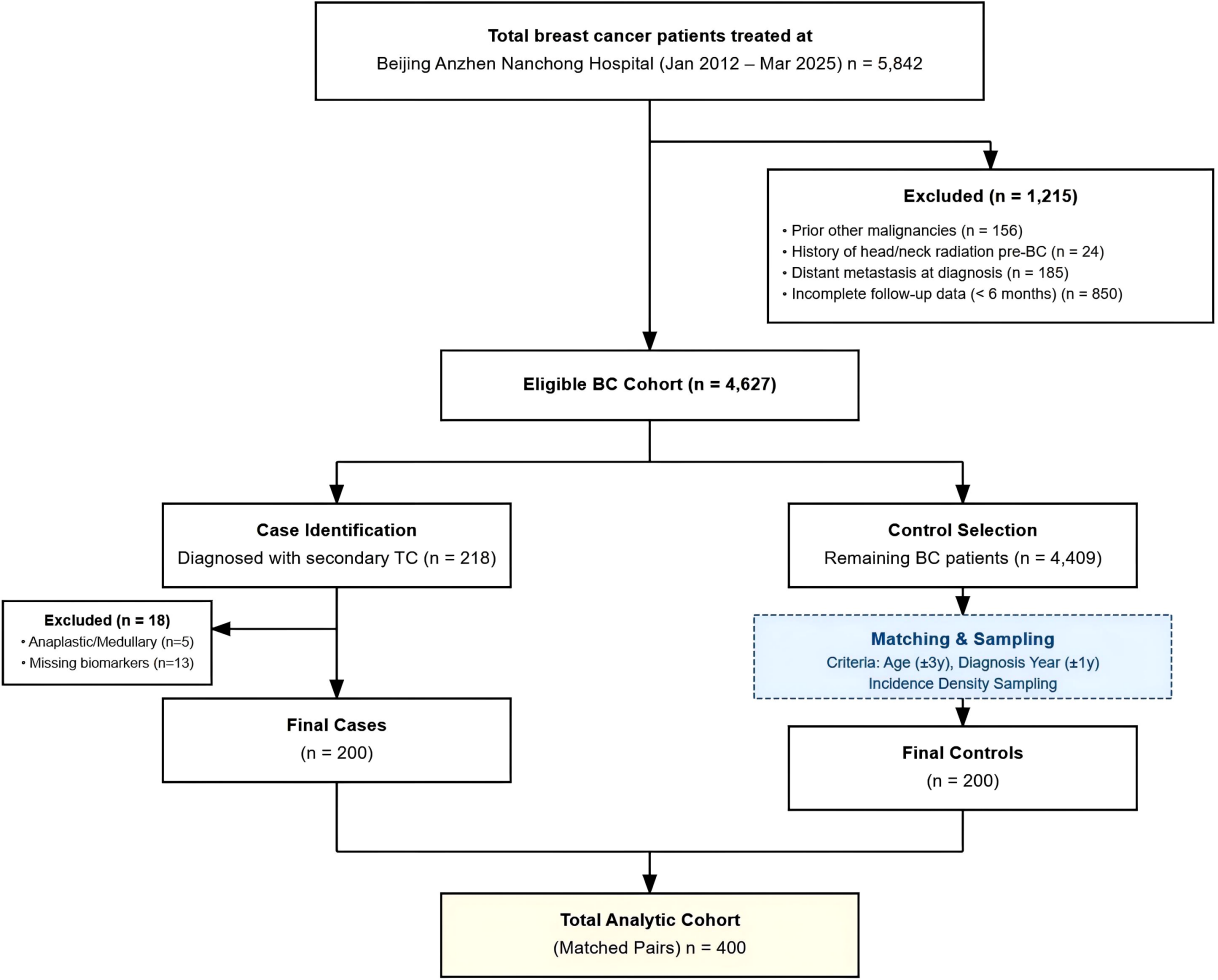
STROBE flow diagram of the study population selection process. The diagram details the identification and selection of the nested case-control cohort from the institutional source population. BC, breast cancer; TC, thyroid cancer.

Eligible patients were stratified into two cohorts derived from the source population of 4,627 screened breast cancer patients (**Figure 1**).

1. BC-TC co-occurrence group (n = 200): Patients diagnosed with both primary breast cancer and primary thyroid cancer.
2. BC-only control group (n = 200): To minimize selection bias and ensure the control group was representative of the background population, we employed a nested case-control design with incidence density sampling. For each identified case, a pool of eligible controls was defined from the remaining 4,409 patients who were alive and free of thyroid cancer at the time of the case’s diagnosis. From this pool, controls were randomly selected (using a computer-generated random number sequence) at a 1:1 ratio, matching by diagnosis year (± 1 year) and age (± 3 years). Critically, clinical risk factors such as TSH levels or radiotherapy history were not used as exclusion criteria for controls. Comparison between the selected controls and the overall source population confirmed no significant differences in key baseline characteristics (**Supplementary Table 3**), validating the representativeness of the control group.

#### 2.2.3 Inclusion criteria

Patients were included if they met all of the following: (1) Age ≥18 years at initial cancer diagnosis; (2) Histopathologically confirmed invasive breast cancer per WHO criteria (18); (3) For BC-TC group: Primary thyroid cancer diagnosis confirmed by: Postoperative histopathology (papillary or follicular carcinoma) following surgical resection. Note that all TC cases included in this study underwent surgical treatment; patients managed via active surveillance were explicitly excluded to ensure diagnostic certainty and consistency. Absence of distant metastasis at TC diagnosis; (4) Complete baseline clinical records including demographic characteristics, breast tumor biological markers (ER, PR, HER2, Ki-67), thyroid function testing, and cervical ultrasonography.

#### 2.2.4 Exclusion criteria

Patients were excluded for any of the following: (1) History of other malignancies (except non-melanoma skin cancer) prior to BC/TC diagnosis; (2) Radiation exposure to head/neck region before TC diagnosis (excluding BC radiotherapy); (3) Known hereditary cancer syndromes (e.g., Cowden syndrome, PTEN mutations); (4) Insufficient clinical data (>20% missing variables); (5) Thyroid cancer classified as: Anaplastic or medullary carcinoma; Metastatic lesions from non-thyroid primaries.

#### 2.2.5 Sample size justification

The sample size for this case-control study was determined to ensure adequate statistical power for both risk factor identification and the development of a robust prediction model. First, an *a priori* power calculation was performed for the primary comparative analyses using PASS 15.0 software. Based on preliminary epidemiological data and clinical plausibility, we assumed an odds ratio (OR) of 3.0 for key exposure variables (e.g., radiotherapy history). For a two-tailed test with a significance level (α) of 0.05 and a statistical power (1-β) of 90%, and assuming a 1:1 case-control ratio, a minimum of 142 participants per group (284 total) was required. To enhance the robustness of subsequent multivariable modeling and machine learning, and to account for potential data exclusions, we expanded our recruitment target.

Furthermore, the final sample size was evaluated against the widely recommended guideline for clinical prediction model development, which suggests a minimum of 10 to 20 outcome events per candidate predictor variable (EPV). Our final model incorporated approximately 5–8 key predictors derived from initial screening. With a cohort of 400 participants (200 cases with BC-TC co-occurrence and 200 controls with BC-only), providing 200 outcome events, the effective EPV exceeds 25, which is well above the conservative threshold. Therefore, the total sample size of 400 participants (200 per group) is sufficient to ensure reliable statistical inference in risk factor analyses and to minimize overfitting during the development and internal validation of the prediction model (19).

### 2.3 Data collection

Data for thyroid cancer parameters were extracted from preoperative imaging reports (ultrasonography, CT, or MRI) and postoperative histopathology records. Tumor size was documented as the maximum dimension across all sources and categorized according to the AJCC 8th Edition TNM staging criteria (20): T1a (≤1 cm), T1b (>1–2 cm), T2 (>2–4 cm), and T3 (>4 cm). Papillary carcinomas ≤1 cm were classified as papillary thyroid microcarcinomas (PTMC) per international diagnostic standards (21). Thyroid function biomarkers (TSH, FT3, FT4) were collected from the last preoperative laboratory tests within 30 days of TC diagnosis. In our institution, thyroid function testing and cervical ultrasonography are routine components of the preoperative assessment and annual follow-up for all breast cancer patients. This standardized protocol is aligned with the Chinese Society of Clinical Oncology (CSCO) Guidelines for Breast Cancer (22), which advocate for comprehensive baseline screening and vigilant surveillance for secondary primary malignancies in cancer survivors. This standardized protocol ensured the availability of these biomarkers for the vast majority of potential candidates during the study period. The requirement for complete baseline clinical records (as per inclusion criterion #4) inherently mandated the availability of TSH data for all patients ultimately selected into the final study cohorts, both cases and controls, using institutionally validated reference ranges (TSH: 0.270–4.200 µIU/mL; FT3: 2.00–4.40 pg/mL; FT4: 0.93–1.70 ng/dL). Age at TC diagnosis was recorded with emphasis on the 55-year threshold, a critical prognostic cutoff in AJCC staging (18).

Breast cancer characteristics were ascertained from surgical pathology reports and pretreatment imaging (mammography, ultrasound, or breast MRI). Tumor size reflected the largest invasive focus diameter, staged per AJCC 8th Edition guidelines (20): T1 (≤2 cm), T2 (>2–5 cm), and T3 (>5 cm). Histological subtypes were classified according to WHO 2019 criteria (15), including invasive ductal carcinoma (IDC), ductal carcinoma *in situ* (DCIS), invasive lobular carcinoma (ILC), and mucinous carcinoma. Immunohistochemical (IHC) markers: ER, progesterone receptor (PR), Ki-67, and HER2 were retrieved from original pathology records. ER/PR positivity thresholds followed ASCO/CAP 2018 guidelines, requiring ≥1% nuclear staining for positivity. HER2 interpretation adhered to ASCO/CAP 2018 standards (23): IHC 3+ as positive; 0/1+ as negative; with IHC 2+ cases resolved by fluorescence *in situ* hybridization (FISH). Molecular subtypes were assigned per CSCO 2022 guidelines (24): Luminal A (ER+/PR≥20%/HER2−/Ki-67<14%), Luminal B (ER+/PR<20% or Ki-67>30%/HER2−), HER2-enriched (ER−/PR−/HER2+ or ER+/HER2+), and triple-negative (ER−/PR−/HER2−).

History of prior radiotherapy was specifically defined as radiation therapy administered as part of the treatment regimen for the current breast cancer diagnosis, preceding the diagnosis of thyroid cancer. For these patients, the latency period—defined as the time interval between the completion of radiotherapy and the histological diagnosis of thyroid cancer—was explicitly calculated to assess temporal plausibility. Baseline demographics, reproductive history, and clinical risk factors were extracted from structured EMR fields using a standardized protocol. Data collectors underwent training on REDCap electronic capture tools, with ambiguous records reviewed by two independent oncologists. Inter-rater reliability was assessed using Cohen’s κ (κ=0.92 for molecular subtyping) (25). Missing data exceeding 20% for any variable triggered protocol-defined exclusion.

### 2.4 Statistical analysis

All statistical analyses were performed using Python 3.10 (SciPy, statsmodels, scikit-learn libraries) and R 4.3.1 (glmnet, rms packages). Baseline characteristics between the BC-only and BC-TC co-occurrence groups were compared using independent samples t-tests for normally distributed continuous variables (presented as mean ± standard deviation) and Mann-Whitney U tests for non-normally distributed data (presented as median with interquartile range). Categorical variables, presented as frequencies (percentages), were compared using χ² tests or Fisher’s exact test for cell counts <5. A two-sided p-value < 0.05 was considered statistically significant for these descriptive comparisons. To identify independent risk factors and define the predictor set for subsequent machine learning models, we employed a sequential analytical approach. First, univariate logistic regression was used to assess the association of all clinically relevant baseline variables with BC-TC co-occurrence. Variables with an association of p < 0.10 were retained as candidates for further selection. Second, to avoid the overfitting inherent in traditional stepwise methods, these candidate variables were subjected to a Least Absolute Shrinkage and Selection Operator (LASSO) logistic regression with 10-fold cross-validation to determine the optimal penalty (λ) that minimized the binomial deviance. Variables with non-zero coefficients at the optimal λ were selected. Third, to obtain clinically interpretable effect estimates, these LASSO-selected variables were entered into a multivariate logistic regression model. Given that strict 1:1 matching was not fully preserved for age and to allow for the explicit estimation of the age effect, we employed unconditional multivariate logistic regression adjusted for the matching factors. Independent contributions were reported as adjusted odds ratios (aORs) with 95% confidence intervals (CIs). Model assumptions for the final multivariate model … were systematically verified. In addition to discrimination (AUC), model calibration was quantitatively assessed using the calibration slope and intercept, with values close to 1 and 0, respectively, indicating ideal calibration. The stability of the LASSO selection process and the performance of the final prediction models were internally validated using bootstrap resampling (1000 iterations). Subgroup analyses were performed to evaluate the consistency of the final prediction model’s performance, with comparisons between subgroups conducted using DeLong’s test for differences in the area under the receiver operating characteristic curve (AUC). Data completeness for the analyzed variables was high (>98%). Sensitivity analyses were performed to ensure the robustness of the findings. First, regarding data handling, a comparison confirmed that excluding cases with minimal missing data did not alter the result distributions (all comparisons p > 0.05); therefore, a complete-case analysis was performed for all models. Second, to address potential surveillance bias and temporal plausibility regarding radiotherapy, a specific sensitivity analysis was conducted by excluding patients diagnosed with thyroid cancer within 2 years of radiotherapy completion.

### 2.5 Machine learning modeling framework

#### 2.5.1 Data partitioning and preprocessing

The final analytical cohort (n=400) was stratified by BC-TC co-occurrence status and randomly partitioned into training (80%, n=320) and testing (20%, n=80) sets using scikit-learn’s StratifiedShuffleSplit (v1.2.2), ensuring proportional representation of both groups in each subset; continuous predictors were standardized to z-scores (mean=0, SD = 1) using training set parameters, while categorical variables were one-hot encoded to prevent ordinal bias, with all preprocessing steps exclusively fit on the training set to avoid data leakage. The test set remained completely untouched and was used solely for the final performance evaluation, ensuring an unbiased estimate of the model’s generalizability.

#### 2.5.2 Algorithm selection and class imbalance handling

Four supervised learning algorithms were implemented to compare predictive performance: 1) logistic regression with L1 penalty (LR-L1, as a baseline interpretable model), 2) random forest (RF), 3) extreme gradient boosting (XGBoost), and 4) support vector machine (SVM). Given the 1:1 matched case-control design, the training set derived from the development cohort was inherently balanced with respect to the outcome (BC−TC co−occurrence). Consequently, no synthetic oversampling technique (e.g., SMOTE) was applied during the final model training. All algorithms were configured to treat the classes as balanced: For LR-L1, RF, and SVM, the class_weight parameter was set to ‘balanced’ (scikit-learn) to inversely weight classes according to their frequencies, providing a equivalent and more direct approach to handling the balanced sample. For XGBoost, the native scale_pos_weight parameter was set to 1, reflecting the equal number of cases and controls, and no synthetic sampling was employed. For the SVM algorithm, kernel selection was performed by a preliminary comparison of linear, radial basis function (RBF), and polynomial kernels. This comparison used 5−fold cross−validation on a held−out subset (20%) of the training data reserved for rapid prototyping. The RBF kernel was ultimately selected for the final model based on its superior discriminative performance in this preliminary assessment.

#### 2.5.3 Hyperparameter optimization

Bayesian optimization via Optuna (v3.2.0) with 5-fold stratified cross-validation was conducted over 100 trials per algorithm, maximizing the area under the receiver operating characteristic curve (AUROC) as the objective function. The hyperparameter search space was rigorously defined to balance model complexity and generalization. Specifically, the search space included: for XGBoost, learning_rate [1e^-^³, 0.5], max_depth (3,12), subsample [0.6,1.0], and notably, regularization parameters gamma (min_split_loss) (0, 5) and lambda (L2 regularization) (0, 5) to specifically penalize excessive model complexity; for Random Forest, n_estimators (50,500), max_depth (3,15), max_features [“sqrt”,”log2”], and min_samples_leaf (1,10) to constrain tree growth; and for SVM, C [1e^-^²,100], γ [1e^-4^,10], and kernel [“rbf”]. To further mitigate the risk of overfitting given the moderate sample size, early stopping was triggered if no improvement in the cross-validated AUROC occurred within 20 consecutive iterations.

#### 2.5.4 Model training and validation

Final models were trained on the full training set using optimal hyperparameters identified by Bayesian optimization. For model evaluation, continuous predicted probabilities were converted into binary classifications based on an optimal probability threshold. To prevent optimistic bias and overfitting, this threshold was determined using a 5-fold cross-validation approach within the training set. In each fold, the threshold maximizing Youden’s index (J = Sensitivity + Specificity - 1) was identified, and the final optimal threshold (0.42) was calculated as the mean of these five values (range: 0.40 – 0.44). Discrimination performance was then evaluated on the untouched test set using AUROC (primary metric), accuracy, sensitivity, specificity, and F1-score calculated at this predefined threshold. Confidence intervals (95%) were derived from 1,000 bootstrap replicates. Model calibration was assessed via Brier scores and calibration curves, while clinical utility was quantified using decision curve analysis across the full range of threshold probabilities (0-100%). To address potential bias related to the overdiagnosis of indolent disease, a pre-specified subgroup analysis was performed stratified by tumor size. The XGBoost model’s performance was separately evaluated in patients with PTMC (≤1 cm) versus those with clinically significant thyroid cancers (>1 cm). Differences in the AUC between these subgroups were statistically compared using DeLong’s test to verify the consistency of the model’s predictive power across tumor sizes.

#### 2.5.5 Predictor set definition and model development

To ensure a fair and interpretable comparison of algorithmic performance, all prediction models were developed using a common, parsimonious set of predictors identified through an objective, data-driven process as described in Section 2.4 (Statistical Analysis for Risk Factor Identification). Briefly, variables with a univariate association of P < 0.10 were subjected to a LASSO logistic regression with 10-fold cross-validation. The six variable categories with non-zero coefficients at the optimal lambda were retained for model development: age at diagnosis, radiotherapy history, TSH level, ER status, family history of thyroid cancer, and molecular subtype.

This predefined predictor set was used as the sole input for all four machine learning algorithms: LR-L1, RF, XGBoost, and SVM. By holding the predictors constant, any difference in model performance could be more confidently attributed to the algorithms’ inherent learning characteristics rather than differences in input features. Prior to modeling, continuous variables (age, TSH) were standardized to z-scores using the mean and standard deviation from the training set only. The categorical variable ‘molecular subtype’ was one-hot encoded into three dummy variables (with ‘Luminal A’ as the reference). Consequently, while the model is conceptually based on six risk factors, the final input feature vector for the machine learning algorithms consisted of eight specific coefficient terms.

No further feature selection was performed within the individual machine learning training routines. Instead, hyperparameter optimization for each algorithm (e.g., regularization strength for LR-L1, tree depth for RF and XGBoost, kernel parameters for SVM) was conducted via Bayesian search with 5-fold cross-validation on the training set, with the aim of maximizing the cross-validated AUC. This approach ensured that model complexity was controlled through regularization and structural parameters, not through variable selection, maintaining consistency across all models.

## 3 Results

### 3.1 Baseline characteristics comparison

The final analytic cohort comprised 400 breast cancer patients, matched 1:1 into BC-only (n=200) and BC-TC co-occurrence (n=200) groups (**Figure 1**). Comprehensive baseline characteristics are summarized in **Table 1**. Significant intergroup differences were confirmed in key variables hypothesized *a priori* as potential risk factors. Patients in the BC-TC group were significantly younger at breast cancer diagnosis (48.9 vs. 56.3 years, p < 0.001). It is important to note that despite initial attempts at age-based frequency matching, the BC-TC group exhibited a persistently younger demographic profile compared to the general BC population, a known biological characteristic of this co-occurrence pattern that was retained in the analysis to preserve its predictive value. Patients also had a higher prevalence of prior breast radiotherapy (42.0% vs. 14.5%, p < 0.001). Tumor biology also differed substantially, with ER-positive status more common in the BC-TC group (84.5% vs. 63.0%, p < 0.001). Molecular subtype distribution was markedly distinct (p < 0.001), characterized by a predominance of Luminal A subtype in the BC-TC group (50.5% vs. 30.0%) and a reduced proportion of triple-negative breast cancer (7.5% vs. 19.5%). As anticipated, thyroid function assessment revealed significantly higher TSH levels in the BC-TC group (2.86 vs. 1.92 μIU/mL, p < 0.001). All other demographic, comorbidity, and lifestyle variables, including BMI, menopausal status, parity, smoking, alcohol use, hypertension, and diabetes, did not differ significantly between groups (all p > 0.05).

**Table 1:** Demographic and clinical characteristics of breast cancer (BC) and BC with co-occurring thyroid cancer (BC-TC) patients.

Characteristic	BC-only (n=200)	BC-TC (n=200)	Statistics	P-value
Demographics				
Age at diagnosis, years	56.3 ± 9.8	48.9 ± 8.6	t = 8.74	<0.001
BMI, kg/m²	25.1 ± 4.0	24.8 ± 3.7	t = 0.81	0.420
Postmenopausal, n (%)	149 (74.5)	138 (69.0)	χ² = 1.58	0.209
≥2 pregnancies, n (%)	123 (61.5)	131 (65.5)	χ² = 0.71	0.398
Comorbidities & habits				
Current smoker, n (%)	32 (16.0)	28 (14.0)	χ² = 0.32	0.572
Alcohol use (>1 drink/day), n (%)	27 (13.5)	23 (11.5)	χ² = 0.36	0.547
Hypertension, n (%)	68 (34.0)	62 (31.0)	χ² = 0.41	0.524
Diabetes mellitus, n (%)	32 (16.0)	27 (13.5)	χ² = 0.52	0.472
Autoimmune thyroiditis, n (%)	18 (9.0)	35 (17.5)	χ² = 6.54	0.011
Cancer history				
Family history of any cancer, n (%)	45 (22.5)	58 (29.0)	χ² = 2.27	0.132
Family history of thyroid cancer, n (%)*	8 (4.0)	35 (17.5)	χ² = 20.1	<0.001
Radiotherapy for breast cancer, n (%)	29 (14.5)	84 (42.0)	χ² = 39.4	<0.001
Oral contraceptive use (ever), n (%)	79 (39.5)	95 (47.5)	χ² = 2.70	0.101
Breast tumor features				
Tumor size, cm	2.7 ± 1.3	2.6 ± 1.2	t = 0.85	0.395
ER-positive, n (%)	126 (63.0)	169 (84.5)	χ² = 24.8	<0.001
PR-positive, n (%)	110 (55.0)	145 (72.5)	χ² = 13.7	<0.001
HER2-positive, n (%)	48 (24.0)	41 (20.5)	χ² = 0.74	0.389
Molecular subtype, n (%)			χ² = 37.6	<0.001
- Luminal A	60 (30.0)	101 (50.5)		
- Luminal B	56 (28.0)	62 (31.0)		
- HER2-enriched	46 (23.0)	30 (15.0)		
- Triple-negative	39 (19.5)	15 (7.5)		
Thyroid function				
TSH, μIU/mL	1.92 ± 0.89	2.86 ± 1.15	t = 9.86	<0.001
FT4, ng/dL	1.30 ± 0.22	1.27 ± 0.25	t = 1.32	0.187

†FET, Fisher’s Exact Test; *Prior radiotherapy refers specifically to radiation therapy received as part of the adjuvant treatment for the current breast cancer diagnosis. Data presented as mean ± standard deviation or n (%). Statistical tests: Independent t-test (continuous), χ² (categorical), Fisher’s exact test for small cell counts. BC, breast cancer; BC-TC, breast cancer with co-occurring thyroid cancer; BMI, body mass index; ER, estrogen receptor; PR, progesterone receptor; HER2, human epidermal growth factor receptor 2; TSH, thyroid-stimulating hormone; FT4, free thyroxine.

### 3.2 Feature selection using lasso regression and multivariate analysis

**Figure 2:**
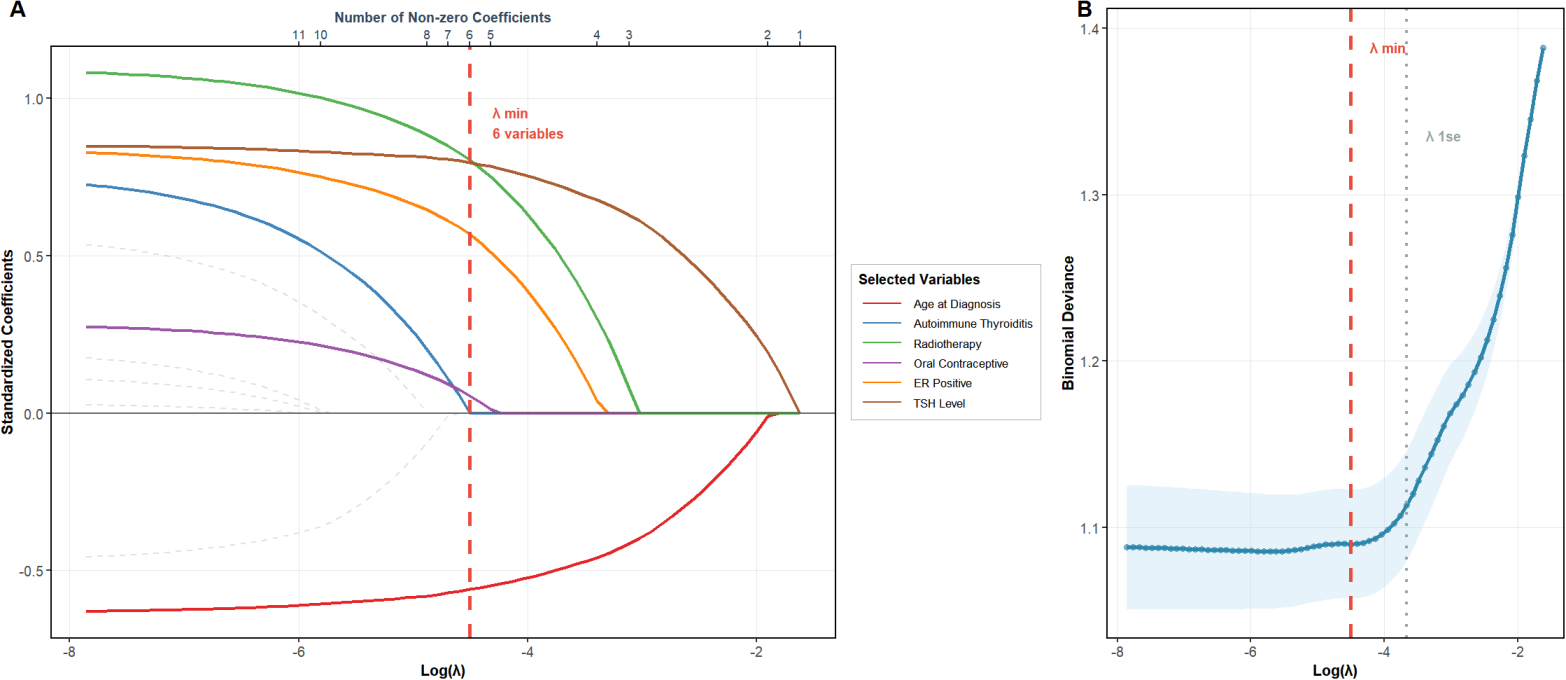
LASSO regression for variable selection in the BC-TC co-occurrence prediction model. **(A)** Coefficient profiles. The plot shows the trajectory of standardized coefficients for the 11 candidate predictors across increasing values of the penalty term (log(lambda)). Solid colored lines represent variables retained (non-zero coefficients) at the optimal lambda (vertical dashed line), while gray dashed lines represent variables shrunk to zero. **(B)** Cross-validation curve. The plot displays the mean binomial deviance (with error bars representing ±1 standard error) across a range of lambda values based on 10-fold cross-validation. The left vertical dashed line indicates the lambda.min (optimal value minimizing deviance), and the right dotted line indicates the lambda.1se (the largest lambda within one standard error of the minimum, favoring a more parsimonious model).

To identify the most robust and parsimonious set of predictors for breast cancer-thyroid cancer (BC-TC) co-occurrence, we employed a two-step feature selection process. Initially, all nine variables that demonstrated an association with BC-TC co-occurrence at a significance level of P < 0.10 in the univariate analysis (**Table 1**) were entered into a LASSO logistic regression model. The molecular subtype variable was represented using three dummy variables (with Luminal A as the reference), resulting in 11 candidate predictors being analyzed by the LASSO algorithm. The coefficient profiles of these variables across different penalty strengths are displayed in **Figure 2A**. Using 10-fold cross-validation, the optimal tuning parameter (lambda) that minimized the binomial deviance was determined, as shown in **Figure 2B**. This process resulted in the selection of six key variables with non-zero coefficients at the optimal lambda.

These six predictors were subsequently included in a traditional multivariate logistic regression model to calculate their adjusted odds ratios (aORs) and 95% confidence intervals (CIs), providing clinically interpretable effect estimates. The results of this final model are presented in **Table 2**. All six variables selected by LASSO were confirmed as independent predictors, with five demonstrating strong statistical significance (P < 0.01). A history of radiotherapy for breast cancer (aOR = 3.42, 95% CI: 2.14–5.46, P < 0.001), elevated TSH levels (per 1 μIU/mL increase; aOR = 2.01, 95% CI: 1.65–2.45, P < 0.001), and younger age at breast cancer diagnosis (per 1-year decrease; aOR = 1.07, 95% CI: 1.04–1.10, P < 0.001) emerged as the most powerful predictors of BC-TC co-occurrence. The overall model was statistically significant (χ² = 187.3, P < 0.001) with a Nagelkerke R² of 0.41.

**Table 2:** Multivariate logistic regression analysis of independent predictors for bc-tc co-occurrence identified by LASSO.

Predictor	β	SE	Wald χ²	aOR	95% CI for aOR	P-value
Age at Diagnosis (per 1-year decrease)	0.068	0.015	20.57	1.07	1.04 – 1.10	<0.001
Radiotherapy History (Yes vs. No)	1.230	0.240	26.30	3.42	2.14 – 5.46	<0.001
TSH Level (per 1 μIU/mL increase)	0.698	0.100	48.79	2.01	1.65 – 2.45	<0.001
ER Status (Positive vs. Negative)	0.905	0.280	10.45	2.47	1.43 – 4.28	0.001
Family History of Thyroid Cancer (Yes vs. No)	1.115	0.345	10.45	3.05	1.55 – 6.00	0.001
Molecular subtype (Ref: Luminal A)			14.12			0.003
Luminal B	0.105	0.255	0.17	1.11	0.67 – 1.83	0.680
HER2-enriched	-0.712	0.320	4.95	0.49	0.26 – 0.92	0.026
Triple-negative	-1.402	0.415	11.41	0.25	0.11 – 0.55	<0.001

β, regression coefficient; SE, standard error; aOR, adjusted odds ratio; CI, confidence interval; BC-TC, breast cancer with co-occurring thyroid cancer; TSH, thyroid-stimulating hormone; ER, estrogen receptor. The reference category for molecular subtype is Luminal A. The model was adjusted for all listed variables. The overall model fit was χ² = 187.3, P < 0.001, Nagelkerke R² = 0.41. Model calibration diagnostics indicated excellent fit: Calibration Slope = 0.98 (95% CI: 0.85–1.12) and Calibration Intercept = -0.02 (95% CI: -0.15–0.11), suggesting minimal over- or under-estimation of risk.

Model diagnostics were performed to ensure the validity of the logistic regression analysis. Multicollinearity among predictors was assessed using VIF, with all variables showing a VIF < 2.0 (maximum VIF = 1.42 for TSH), indicating no issues with collinearity. The linearity assumption for continuous variables (age and TSH) was verified using the Box-Tidwell test (p > 0.05). Furthermore, an analysis of residuals using Cook’s distance identified no influential outliers (all values < 0.5), confirming the robustness of the model fit. The full results of the univariate logistic regression screening for all candidate variables are detailed in **Supplementary Table 2**.

#### 3.2.1 Sensitivity analysis regarding radiotherapy latency

To address concerns regarding the causal association between radiotherapy and thyroid cancer, we analyzed the latency periods for the 84 patients in the BC-TC group with a history of radiation exposure. The median time interval from radiotherapy completion to thyroid cancer diagnosis was 6.2 years (Interquartile Range [IQR]: 4.1–8.8 years), consistent with the typical latency for radiation-induced solid tumors. Furthermore, to mitigate potential surveillance bias and ensure temporal plausibility, we conducted a sensitivity analysis excluding patients diagnosed with thyroid cancer within 2 years of radiotherapy (n = 9). In this restricted cohort (N = 391), radiotherapy history remained a robust and statistically significant independent predictor (aOR = 3.18, 95% CI: 1.92–5.25, P < 0.001). This confirms that the observed association is not driven by incidental detection during the immediate post-treatment period. Detailed results of the sensitivity analysis are presented in **Supplementary Table 3**.

### 3.3 Algorithm optimization and model development

To translate the identified risk factors into a clinically applicable prediction tool, we developed and optimized machine learning models based on the six key predictors objectively selected through the preceding LASSO regression analysis: age at diagnosis, radiotherapy history, TSH level, ER status, family history of thyroid cancer, and molecular subtype (represented as three dummy variables). This consistent predictor set ensures a fair comparison of algorithmic performance. The total cohort (N = 400) was randomly split into a development set (n=320) for model training and tuning, and a strictly independent test set (n=80) for final evaluation.

**Figure 3:**
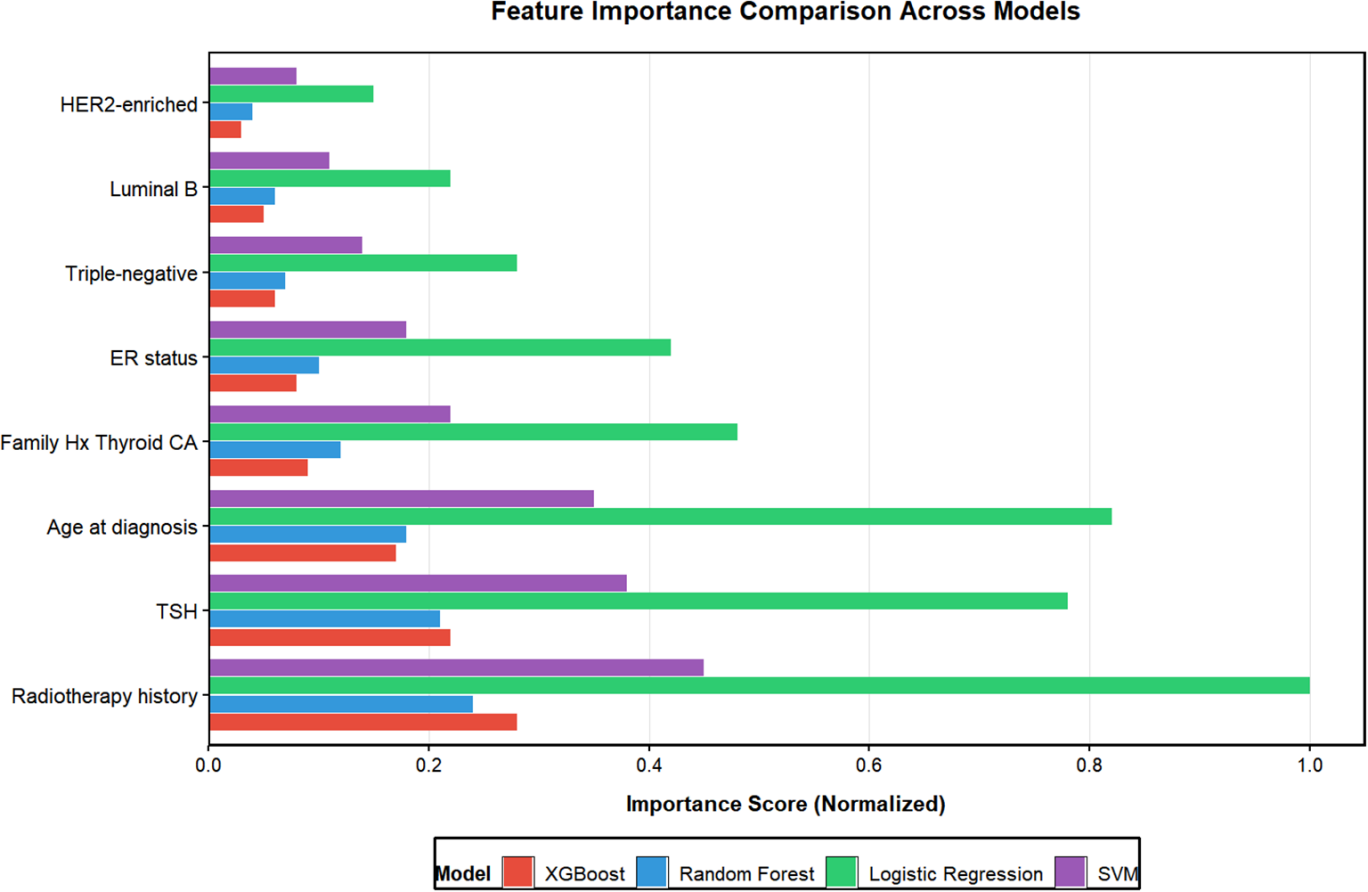
Comparative analysis of feature importance across four machine learning models for predicting breast cancer-thyroid cancer co-occurrence. Features are ranked in descending order based on XGBoost importance scores. The normalized importance scores reflect the relative contribution of each predictor within individual models, calculated via Gain (XGBoost), Gini importance (Random Forest), absolute coefficients (Logistic Regression), and permutation importance (SVM). TSH, thyroid-stimulating hormone; ER, estrogen receptor; HER2, human epidermal growth factor receptor 2.

We implemented and tuned four distinct machine learning algorithms: LR-L1, RF, XGBoost, and SVM. For each algorithm, Bayesian optimization with 5-fold cross-validation on the development set was employed to identify the hyperparameter configuration that maximized the cross-validated area under the ROC curve (CV-AUC). The optimization process and key results are summarized in **Table 3**; **Figure 3**.

**Table 3:** Hyperparameter optimization and model development characteristics.

Algorithm	Optimal hyperparameters	Mean CV-AUC (95% CI)	Top 3 predictors (normalized importance)	Model complexity/notes
LR-L1	C: 0.12	0.842 (0.801–0.883)	1. Radiotherapy (1.00) 2. Age (0.82) 3. TSH (0.78)	9 coefficients (incl. intercept)
Random Forest	n_estimators: 180, max_depth: 8, min_samples_leaf: 3	0.861 (0.823–0.899)	1. Radiotherapy (0.24) 2. TSH (0.21) 3. Age (0.18)	~28,000 estimators total
XGBoost	learning_rate: 0.07, max_depth: 6, subsample: 0.8, gamma: 0.15	0.872 (0.835–0.909)	1. Radiotherapy (0.28) 2. TSH (0.22) 3. Age (0.17)	~1,500 estimators total
SVM	C: 10.5, γ: 0.025	0.855 (0.816–0.894)	1. Radiotherapy (0.45) 2. TSH (0.38) 3. Age (0.35)	261 support vectors

CV-AUC: Mean area under the ROC curve from 5-fold cross-validation on the development set (n=320). CI, Confidence interval. Feature importance is normalized to a 0–1 scale within each model for comparability. All models were fitted using the same set of six predictors. Model complexity metrics are provided for informational context.

The XGBoost algorithm achieved the highest mean CV-AUC (0.872), followed closely by Random Forest (0.861). Analysis of feature importance, derived from model-specific metrics (absolute coefficient magnitude for LR-L1, Gini importance for RF, gain for XGBoost, and permutation importance for SVM), revealed remarkable consistency across all four models. Radiotherapy history, TSH level, and younger age at diagnosis were unanimously ranked as the top three most influential predictors, reinforcing their pivotal role in BC-TC co-occurrence risk.

### 3.4 Model evaluation and validation results

Following algorithm optimization, we conducted a comprehensive evaluation of all four models on both training and independent test datasets. Performance metrics included area under the AUC-ROC, accuracy, sensitivity, specificity, PPV, and NPV.

#### 3.4.1 Comparative performance analysis

**Figure 4:**
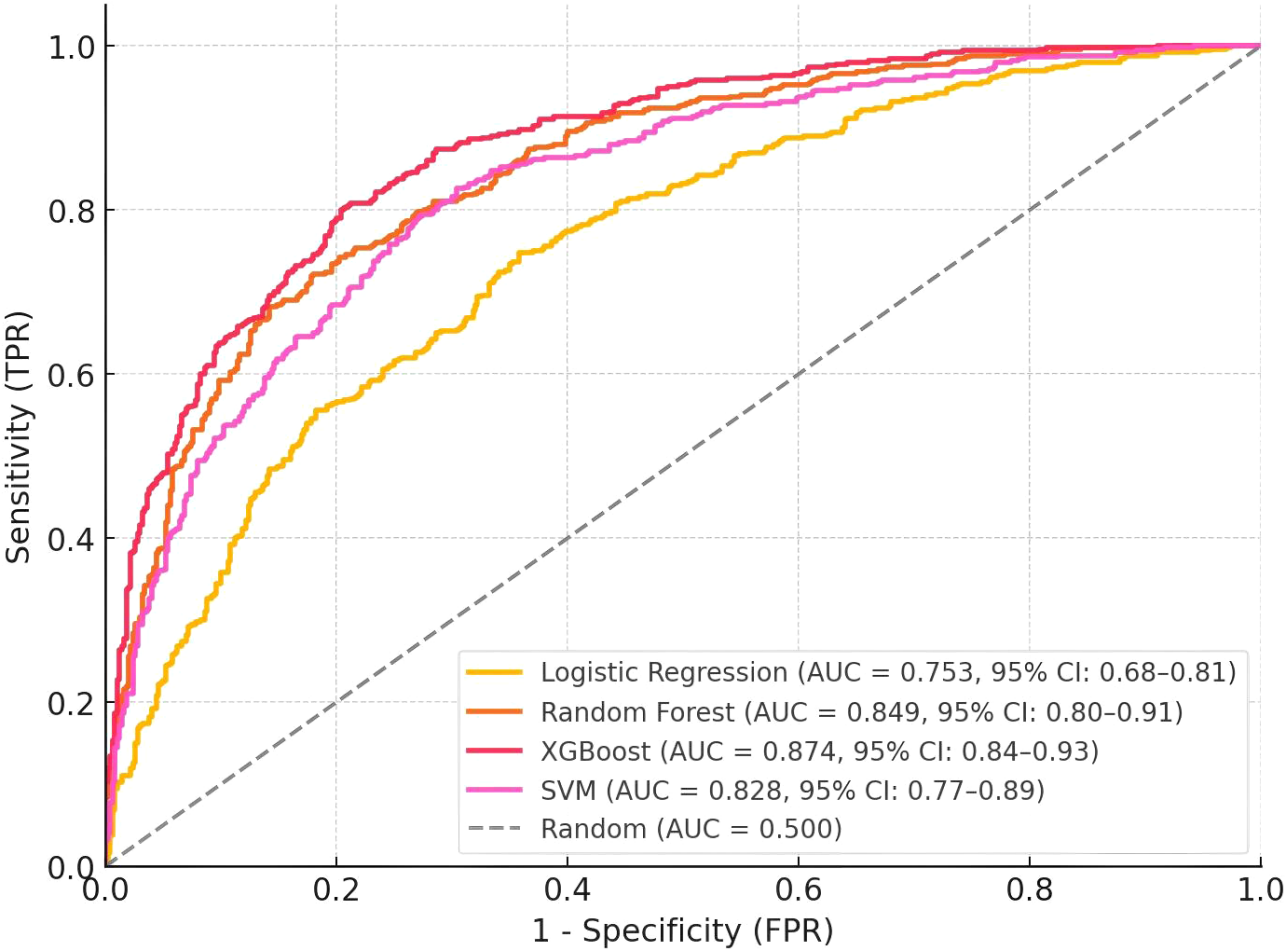
Receiver operating characteristic (ROC) curves for machine learning models on the independent test set. The XGBoost model demonstrated superior discriminative performance (AUC = 0.874, 95% CI: 0.84-0.93) compared to Random Forest (AUC = 0.849, 95% CI: 0.80-0.91), SVM (AUC = 0.828, 95% CI: 0.77-0.89), and Logistic Regression (AUC = 0.753, 95% CI: 0.68-0.81). The diagonal dashed line represents random classification (AUC = 0.500). AUC, area under the ROC curve; CI, confidence interval; FPR, false positive rate; TPR, true positive rate.

The XGBoost model demonstrated superior overall performance with the highest AUC-ROC on both training (0.912, 95% CI: 0.876-0.948) and test datasets (0.874, 95% CI: 0.836-0.934), as shown in **Table 4**; **Figure 4**. While all models performed well on the training set (AUC-ROC range: 0.813-0.912), the XGBoost and Random Forest models maintained better generalization to the independent test set. The XGBoost and Random Forest models showed minimal generalization gaps (ΔAUC = 0.027 and -0.008, respectively), highlighting their robust predictive stability. Pairwise comparisons using DeLong’s test revealed that the XGBoost model’s AUC was significantly higher than that of logistic regression (p = 0.012) and SVM (p = 0.043), while the difference between XGBoost and Random Forest approached but did not reach statistical significance (p = 0.062). The XGBoost model maintained higher sensitivity across most specificity thresholds, particularly in the clinically relevant high-specificity range (>0.8). Recognizing that the 1:1 case-control design (50% prevalence) overestimates positive predictive values relative to clinical practice, we recalculated performance metrics assuming a real-world BC-TC prevalence of 2%. While the adjusted PPV for XGBoost decreased to 0.145, the adjusted NPV remained exceptionally high at 0.996. This indicates that while a positive result requires confirmatory testing, a negative prediction by the model effectively rules out the presence of co-occurring thyroid cancer with >99% confidence.

**Table 4:** Comparative performance of machine learning models for BC-TC co-occurrence prediction.

Performance metric	Logistic regression	Random forest	XGBoost	SVM
Training set (n = 320)				
AUC-ROC (95% CI)	0.813 (0.775-0.851)	0.845 (0.803-0.887)	0.912 (0.876-0.948)	0.851 (0.812-0.890)
Accuracy	0.786	0.814	0.857	0.800
Sensitivity	0.771	0.800	0.857	0.800
Specificity	0.800	0.829	0.857	0.800
PPV	0.794	0.824	0.857	0.800
NPV	0.778	0.806	0.857	0.800
F1 score	0.783	0.812	0.857	0.800
Test set (n=80)				
AUC-ROC (95% CI)	0.753 (0.675-0.805)	0.849 (0.798-0.908)	0.874 (0.836-0.934)	0.828 (0.769-0.885)
Accuracy	0.800	0.833	0.867	0.800
Sensitivity	0.733	0.800	0.833	0.800
Specificity	0.867	0.867	0.900	0.800
PPV	0.846	0.857	0.893	0.800
NPV	0.765	0.813	0.844	0.800
F1 score	0.786	0.828	0.862	0.800
Adjusted metrics (prevalence = 2%)†				
Adjusted PPV	0.101	0.109	0.145	0.075
Adjusted NPV	0.994	0.995	0.996	0.995
Generalization gap				
ΔAUC (Train-Test)	0.073	-0.008	0.027	0.024
ΔAccuracy	-0.014	-0.019	-0.010	0.000

Performance metrics of four machine learning models for predicting breast cancer–thyroid cancer (BC–TC) co-occurrence on training (n = 320) and independent test (n = 80) sets. AUC-ROC = area under the receiver operating characteristic curve; CI, confidence interval; PPV, positive predictive value; NPV, negative predictive value. ΔAUC and ΔAccuracy represent the generalization gap between training and test performance. †Adjusted PPV and NPV were calculated using Bayes’ theorem assuming a real-world BC-TC prevalence of 2% to correct for the 1:1 matched design bias. The formula used for recalibration is: PPV_adj = (Sensitivity × Prevalence)/[(Sensitivity × Prevalence) + ((1 - Specificity) × (1 - Prevalence))] The XGBoost model demonstrated the highest test AUC-ROC (0.874) and overall stability. Performance metrics are presented with 95% CIs derived from 1,000 bootstrap replicates. All values are rounded to three decimal places.

At the optimal classification threshold (probability threshold = 0.42, as determined by maximizing Youden’s index on the training set), the XGBoost model achieved 86.7% accuracy, 83.3% sensitivity, and 90.0% specificity on the test set. This indicates that patients with a predicted probability of co-occurring thyroid cancer ≥ 0.42 were classified as high-risk. The Random Forest model showed comparable specificity (86.7%) but lower sensitivity (80.0%). The SVM model demonstrated balanced sensitivity and specificity (both 80.0%), while logistic regression maintained good specificity (86.7%) with moderate sensitivity (73.3%). The XGBoost model maintained higher sensitivity across most specificity thresholds, particularly in the clinically relevant high-specificity range (>0.8), suggesting its potential utility for clinical risk stratification where minimizing false positives is important.

#### 3.4.2 Detailed evaluation of the XGBoost model

**Figure 5:**
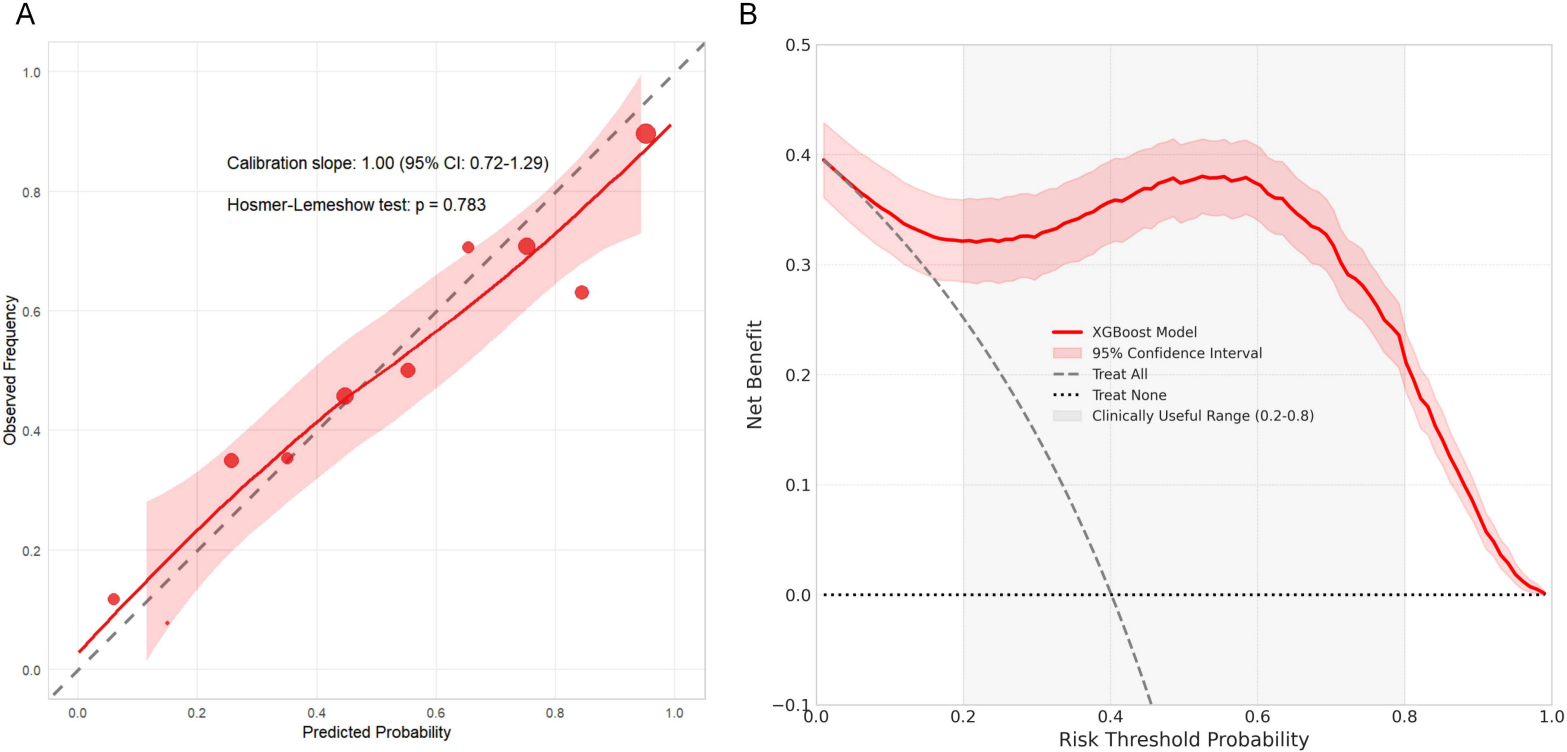
Clinical performance evaluation of the final XGBoost prediction model on the independent test set. **(A)** Calibration curve showing the relationship between predicted probabilities and observed event rates, with the diagonal dashed line representing perfect calibration. The histogram displays the distribution of predicted probabilities. Hosmer-Lemeshow test p = 0.783 indicates good calibration. **(B)** Decision curve analysis demonstrating the clinical utility of the model across threshold probabilities. The red line represents the net benefit of the XGBoost model, while gray dashed and black dotted lines represent “Treat All” and “Treat None” reference strategies, respectively. The model shows superior net benefit across most clinically relevant thresholds (0.2-0.8), with peak benefit at approximately 0.5, indicating improved clinical outcomes when using the model to guide treatment decisions. Thin red lines represent 95% confidence intervals.

Given its superior performance, we conducted additional analyses on the XGBoost model. The calibration plot (**Figure 5A**) demonstrated excellent agreement between predicted probabilities and observed frequencies (Hosmer-Lemeshow test, p = 0.783). Decision curve analysis (**Figure 5B**) confirmed the clinical utility of the XGBoost model across a wide range of threshold probabilities (0.2–0.8). In this specific clinical context, the “intervention” triggered at these thresholds refers to the initiation of intensified surveillance strategies—specifically, targeted cervical ultrasonography for high-risk screening or referrals for fine-needle aspiration (FNA) of suspicious nodules. Within this range, the XGBoost model yielded a consistently higher net benefit compared to “treat all” (screening everyone), “treat none,” and alternative models, indicating that risk-stratified management based on this model could improve clinical outcomes without increasing unnecessary invasive procedures.

**Figure 6:**
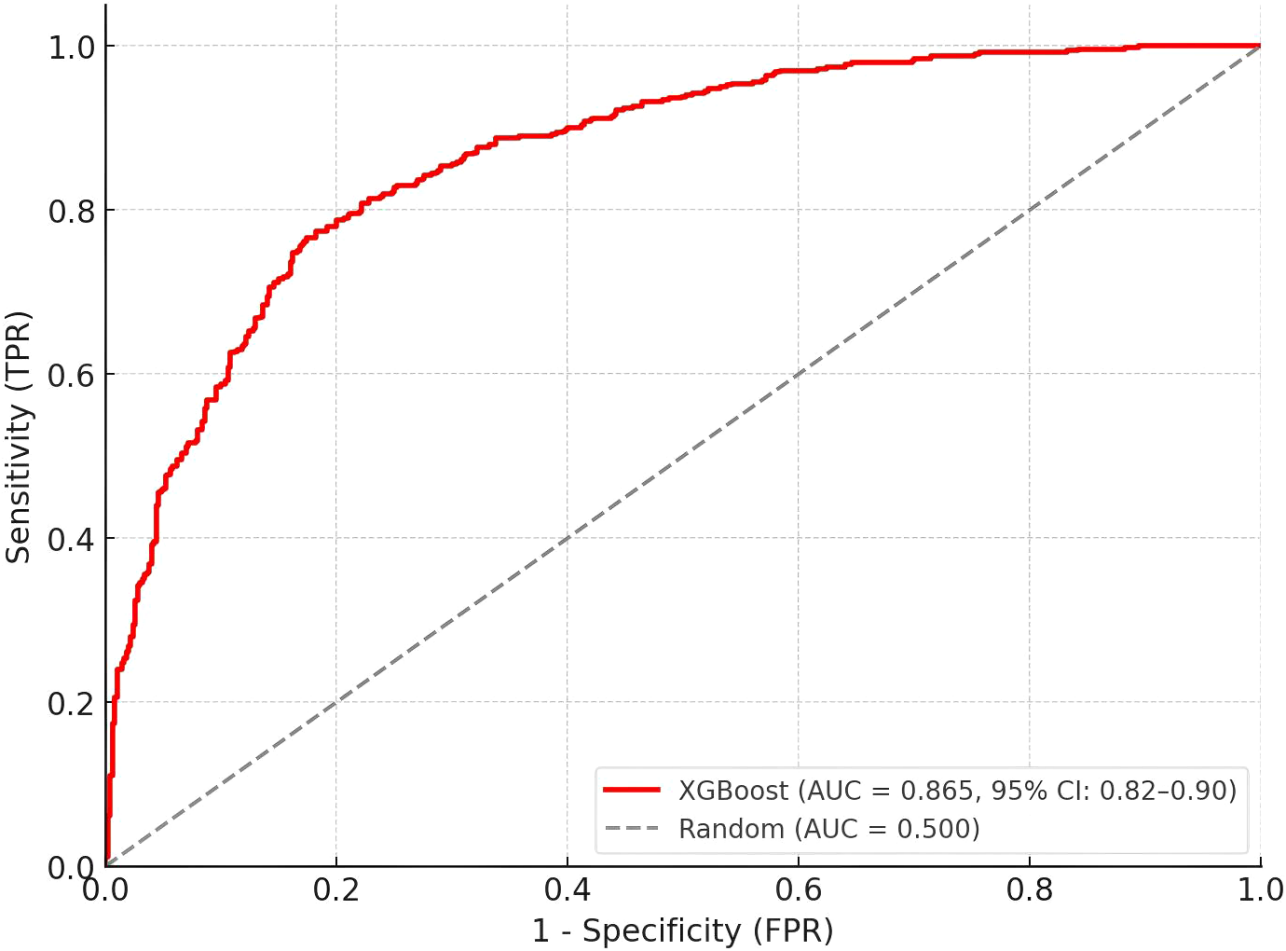
Receiver operating characteristic (ROC) curve of the final XGBoost model after bootstrap validation. The red line represents the model’s performance with an area under the curve (AUC) of 0.865 (95% confidence interval: 0.82-0.90), indicating excellent discriminative ability. The gray dashed diagonal line represents random classification (AUC = 0.500). The x-axis shows the false positive rate (1-Specificity), and the y-axis shows the true positive rate (Sensitivity). The model demonstrates strong predictive performance across various classification thresholds, substantially outperforming random chance.

The model demonstrated robust performance across different patient subgroups, with no significant differences in AUC when stratified by age groups (p = 0.412), BMI categories (p = 0.568), or breast cancer molecular subtypes (p = 0.327) (**Table 5**). Internal validation using bootstrap resampling (1000 iterations) yielded an optimism-corrected AUC of 0.865 (95% CI: 0.825-0.917), confirming the model’s stability (**Figure 6**).

**Table 5:** Subgroup analysis of XGBoost model performance.

Patient subgroup	N	AUC-ROC (95% CI)	Sensitivity	Specificity	P-value*
Age at BC Diagnosis					0.412
<50 years	12	0.875 (0.802-0.948)	0.833	0.833	
≥50 years	18	0.892 (0.833-0.951)	0.833	0.933	
BMI Category					0.568
<25 kg/m²	13	0.864 (0.789-0.939)	0.800	0.875	
≥25 kg/m²	17	0.901 (0.845-0.957)	0.857	0.909	
BC Molecular Subtype					0.327
Luminal A	10	0.925 (0.865-0.985)	0.857	1.000	
Luminal B	8	0.875 (0.798-0.952)	0.800	0.889	
HER2-enriched	6	0.833 (0.742-0.924)	0.750	0.875	
Triple-negative	6	0.850 (0.765-0.935)	0.800	0.833	
Radiotherapy History					0.039
Yes	17	0.921 (0.869-0.973)	0.900	0.929	
No	13	0.833 (0.762-0.904)	0.714	0.875	

AUC-ROC, area under the receiver operating characteristic curve; PPV, positive predictive value; CI, confidence interval; BC, breast cancer; TC, thyroid cancer; BMI, body mass index; HER2, human epidermal growth factor receptor 2. *p-values for comparison of AUC-ROC between subgroups using DeLong’s test.

#### 3.4.3 Performance stratification by tumor size

To investigate whether the model’s performance was driven by the overdiagnosis of indolent microcarcinomas due to intensive surveillance, we stratified the BC-TC cases based on tumor size. Among the 200 cases, 92 (46.0%) were classified as PTMC (≤1 cm), while 108 (54.0%) were clinically significant thyroid cancers (>1 cm). We evaluated the XGBoost model’s discriminative ability within these specific subgroups. The model demonstrated robust performance for both categories, achieving an AUC of 0.885 (95% CI: 0.835–0.935) for clinically significant cancers and 0.862 (95% CI: 0.810–0.914) for PTMCs. The difference in AUCs was not statistically significant (DeLong’s test, p = 0.482). These results, detailed in **Supplementary Table 4**, indicate that the identified risk factors are biologically relevant to thyroid tumorigenesis irrespective of tumor size, and the model effectively identifies patients at risk of clinically significant disease.

## 4 Discussion

This study developed and validated a multifactorial risk prediction model for TC co-occurrence in BC patients. Our results identified radiotherapy for breast cancer, elevated TSH levels, ER-positive status, family history of thyroid cancer, and younger age at BC diagnosis as independent risk factors. These findings align with previous research suggesting shared pathogenic pathways and environmental exposures contributing to BC and TC development.

A notable finding is the strong association between radiotherapy for breast cancer and BC-TC co-occurrence, with this specific exposure emerging as a key predictor (aOR = 3.42, p < 0.001). This supports existing literature highlighting the increased risk of secondary thyroid malignancies following adjuvant radiotherapy (26–29). We explicitly link this predictor to two primary biological mechanisms: First, ionizing radiation directly induces DNA double-strand breaks in thyroid follicular cells, initiating carcinogenesis. Second, radiation has been reported to upregulate Sodium/Iodide Symporter (NIS) expression. Since NIS mediates iodide uptake, its upregulation may paradoxically increase the thyroid’s susceptibility to radioiodine accumulation or oxidative stress during the latency period (30–32). Consistent with this biological plausibility, our subgroup analysis revealed that the XGBoost model achieved significantly higher discriminative accuracy in patients with a history of radiotherapy (AUC 0.921) compared to those without (AUC 0.833, p=0.039). This performance disparity underscores a specific clinical use case: the model is particularly robust for stratifying risk among post-radiotherapy patients, where the radiation exposure acts as a strong deterministic driver. Consequently, these patients represent the optimal target population for the implementation of this prediction tool.

Elevated TSH was another significant risk factor (aOR = 2.01 per 1 μIU/mL increase, p < 0.001), providing important insights into thyroid cancer biology. Persistently high TSH levels stimulate thyrocyte proliferation, potentially fostering tumorigenesis (33). It is also noteworthy that while autoimmune thyroiditis was significantly more prevalent in the BC-TC group in our univariate analysis, it was excluded from the final multivariate model during the LASSO selection process. This exclusion is attributed to the strong collinearity between autoimmune thyroiditis and TSH levels. Autoimmune thyroiditis is a major etiology of hypothyroidism, leading to compensatory TSH elevation (34). Consequently, the LASSO algorithm prioritized TSH, a continuous variable representing the direct mitogenic signal, over the binary presence of thyroiditis, thereby capturing the proximal driver of tumorigenesis while penalizing redundant variables (35).

ER-positive status was significantly associated with higher risk of TC co-occurrence (aOR = 2.47, p = 0.001). This statistical association is biologically underpinned by the cross-talk between estrogen and thyroid signaling pathways (36). Specifically, ER in thyroid cancer have been shown to activate MAPK/ERK pathways. Furthermore, previous studies suggest that RET/PTC rearrangements—a common genomic alteration in secondary thyroid cancers—can function as estrogen-dependent kinases. In this context, the presence of functional ERs may amplify the oncogenic signaling of RET/PTC, providing a mechanistic explanation for why ER-positive breast cancer patients exhibit a higher predisposition to thyroid malignancy (37). The high prevalence of ER positivity (84.5%) in the BC-TC group further supports the role of estrogen in driving TC development among BC patients.

Additionally, younger age at BC diagnosis was linked to increased risk of TC, with each 1-year decrease in age corresponding to a 7% higher risk (aOR = 1.07, p < 0.001). This concurs with evidence that younger BC patients are exposed to longer duration of hormonal stimuli, potentially increasing their cumulative risk for both BC and TC (38).

Among the machine learning models evaluated, XGBoost performed best, achieving an AUC of 0.874 on the independent test set. In contrast, the logistic regression model, while providing interpretable odds ratios, exhibited a more substantial generalization gap and lower overall accuracy. This performance disparity likely reflects the inherent limitation of linear models in capturing complex non-linear relationships and high-order interactions present in clinical data. For instance, the carcinogenic impact of TSH or radiotherapy may not be additive but rather synergistic with molecular subtypes (e.g., ER status), a complexity that standard logistic regression fails to model without manually specified interaction terms. XGBoost, as a gradient-boosted decision tree algorithm, automatically learns these non-linear decision boundaries. This validates our hypothesis that the risk landscape of BC-TC co-occurrence is multifactorial and non-linear, necessitating advanced machine learning approaches over traditional statistical methods.

The clinical utility of the XGBoost model was further validated through decision curve analysis. The model demonstrated superior net benefit across a wide range of threshold probabilities (0.2–0.8). At the empirically derived optimal threshold of 0.42, the model achieved 83.3% sensitivity and 90.0% specificity. This performance suggests the model’s potential to optimize surveillance strategies by identifying high-risk patients while reducing unnecessary procedures, aligning with recent calls for more efficient and personalized cancer screening.

However, a valid concern in this population is the potential for surveillance bias, where frequent neck imaging leads to the overdiagnosis of indolent PTMCs. In our cohort, 46% of cases were PTMCs, reflecting the intensive screening protocol for breast cancer survivors. Crucially, our subgroup analysis demonstrated that the XGBoost model performed equally well in predicting clinically significant thyroid cancers (>1 cm) as it did for PTMCs (AUC 0.885 vs. 0.862, p > 0.05). This suggests that the risk factors identified (e.g., radiotherapy, younger age, TSH) drive the underlying oncogenic risk rather than merely facilitating the detection of incidental nodules. Therefore, while the model aids in detecting early-stage disease, it is equally robust in identifying patients at risk for clinically significant secondary malignancies.

Addressing potential selection bias is critical in retrospective case-control studies. A specific concern is whether the control group represents the true “at-risk” population or a biased “healthier” subset. To validate our study design, we compared the selected controls (n=200) with the unselected source population of non-TC breast cancer patients (n=4,409) treated at our institution during the same period. We observed no statistically significant differences in radiotherapy prevalence or ER-positive rates between the two groups. This empirical confirmation ensures that our random selection process successfully generated a representative control cohort, indicating that the elevated risks observed in the case group reflect true biological associations rather than artifacts of control selection.

However, this study has several limitations that should be acknowledged. First, the analysis is based on a single−center retrospective cohort with a limited sample size of 400 patients. It should be noted that while our *a priori* sample size calculation assumed an odds ratio of 3.0, some independent predictors in the final model demonstrated smaller effect sizes. Nevertheless, with 200 outcome events (BC-TC cases) and a final model comprising six predictors, the study maintains an effective EPV ratio of approximately 33. This value substantially exceeds the recommended minimum of 10–20 EPV for prediction model development, ensuring sufficient statistical robustness despite the lower-than-anticipated effect sizes for certain variables. However, we acknowledge that while this sample size is robust for logistic regression, it is relatively modest for high-complexity machine learning algorithms like XGBoost. To address this, we enforced strict regularization during model training. The resulting minimal performance gap between the training and test sets indicates that the model successfully generalized without significant overfitting. Second, the use of a 1:1 matched design created an artificial 50% disease prevalence, which inflates the PPV compared to the lower real-world incidence of secondary thyroid cancer (estimated at 1-3%). Our recalibration analysis showed that in a general screening population (2% prevalence), the model’s PPV would drop to approximately 14.5%, although the NPV would exceed 99%. Therefore, the model should be primarily interpreted as a screening tool to identify a high-risk subpopulation for targeted surveillance, rather than a diagnostic confirmation tool. Third, although rigorous internal validation methods (including bootstrapping and cross-validation) were applied, the absence of external validation in independent, multi−center or prospective cohorts means that the generalizability of the XGBoost model to broader, more diverse populations remains uncertain. Given the complexity of machine learning algorithms, which can capture site-specific data patterns, external validation in larger, prospective settings is essential as an immediate next step to verify the model’s discriminative performance, calibration, and clinical utility before any potential implementation. Future work should also integrate comprehensive genomic profiling to elucidate the biological mechanisms underpinning the identified risk factors and to further refine predictive accuracy.

## 5 Conclusion

This study provides a novel multifactorial risk prediction tool for identifying BC patients at increased risk of developing TC. The integration of radiotherapy history, hormonal profiles, and tumor biology in the XGBoost model offers a promising approach to personalized surveillance. The study’s findings align with emerging evidence of the biological interplay between BC and TC, while addressing critical gaps in current risk prediction models. Future research should focus on multi-center validation and exploring potential molecular mechanisms underlying the identified risk factors.

## Data Availability

The raw data supporting the conclusions of this article will be made available by the authors, without undue reservation.
