# Supplementary material for: Development and validation of a multifactorial risk prediction model for breast cancer patients with co-occurring thyroid cancer: a retrospective matched case-control study
Source: Front Oncol. 2026 Mar 17;16:1772910. doi: 10.3389/fonc.2026.1772910 (PMC13035759; doi:10.3389/fonc.2026.1772910)
Supplement: Supplementary file 1 [file Table1.docx]

**Supplementary Table S1.** Comparison of clinical characteristics between the randomly selected control group and the eligible source population

| **Characteristic** | **Selected Controls**  **(n=200)** | **Source Population**  **(n=4,409)** | **P-value** |
| --- | --- | --- | --- |
| Age at BC Diagnosis, years (mean ± SD) | 56.3 ± 9.8 | 55.6 ± 10.1 | 0.354 |
| Radiotherapy History, n (%) | 29 (14.5%) | 692 (15.7%) | 0.648 |
| ER-Positive Status, n (%) | 126 (63.0%) | 2,746 (62.3%) | 0.835 |
| Molecular Subtype, n (%) |  |  | 0.741 |
| - Luminal A | 60 (30.0%) | 1,291 (29.3%) |  |
| - Luminal B | 56 (28.0%) | 1,225 (27.8%) |  |
| - HER2-enriched | 46 (23.0%) | 1,023 (23.2%) |  |
| - Triple-negative | 39 (19.5%) | 870 (19.7%) |  |

**Notes:** The 'Source Population' refers to the eligible cohort of breast cancer patients treated at our institution between January 2012 and March 2025 who did not develop secondary thyroid cancer. Data are presented as mean ± standard deviation (SD) for continuous variables and as number (percentage) for categorical variables. P-values were calculated using the independent samples t-test for age and the Pearson's χ² test for categorical variables (radiotherapy history, ER status, and molecular subtype). The non-significant differences across all evaluated characteristics (all p > 0.05) indicate that the randomly selected control group is highly representative of the general institutional source population, confirming the absence of selection bias. Abbreviations: BC, breast cancer; ER, estrogen receptor; HER2, human epidermal growth factor receptor 2; SD, standard deviation.

**Supplementary Table S2.** Univariate logistic regression analysis of predictors for breast cancer-thyroid cancer co-occurrence

| **Variable** | **OR (95% CI)** | **P-value** |
| --- | --- | --- |
| Age at Diagnosis (per 1-year decrease) | 1.09 (1.06 – 1.12) | <0.001 |
| BMI (kg/m²) | 0.98 (0.93 – 1.03) | 0.42 |
| Postmenopausal (Yes vs. No) | 0.76 (0.49 – 1.17) | 0.209 |
| Parity (≥2 vs. <2) | 1.19 (0.79 – 1.78) | 0.398 |
| Current Smoker (Yes vs. No) | 0.86 (0.50 – 1.47) | 0.572 |
| Alcohol Use (Yes vs. No) | 0.83 (0.46 – 1.49) | 0.547 |
| Hypertension (Yes vs. No) | 0.87 (0.57 – 1.34) | 0.524 |
| Diabetes Mellitus (Yes vs. No) | 0.82 (0.48 – 1.41) | 0.472 |
| Autoimmune Thyroiditis (Yes vs. No) | 2.15 (1.18 – 3.92) | 0.011 |
| Family History of Any Cancer (Yes vs. No) | 1.40 (0.90 – 2.18) | 0.132 |
| Family Hx of Thyroid Cancer (Yes vs. No) | 5.09 (2.30 – 11.26) | <0.001 |
| Radiotherapy History (Yes vs. No) | 4.27 (2.65 – 6.89) | <0.001 |
| Oral Contraceptive Use (Yes vs. No) | 1.39 (0.94 – 2.06) | 0.101 |
| Tumor Size (cm) | 0.94 (0.80 – 1.10) | 0.395 |
| ER Status (Positive vs. Negative) | 3.20 (2.02 – 5.08) | <0.001 |
| PR Status (Positive vs. Negative) | 2.14 (1.41 – 3.25) | <0.001 |
| HER2 Status (Positive vs. Negative) | 0.82 (0.51 – 1.31) | 0.389 |
| Molecular Subtype (Ref: Luminal A) |  | <0.001 |
| Luminal B | 0.66 (0.41 – 1.05) | 0.078 |
| HER2-enriched | 0.39 (0.23 – 0.66) | <0.001 |
| Triple-negative | 0.23 (0.12 – 0.44) | <0.001 |
| TSH Level (per 1 μIU/mL increase) | 2.35 (1.92 – 2.88) | <0.001 |
| FT4 Level (ng/dL) | 0.62 (0.31 – 1.25) | 0.187 |

Notes: OR: odds ratio; CI: confidence interval.

**Supplementary Table S3.** Sensitivity analysis of independent predictors for BC-TC co-occurrence excluding cases with short latency (< 2 years) from radiotherapy

| **Predictor** | **Original Model (N=400)** | | **Sensitivity Analysis Model (N=391)†** | |
| --- | --- | --- | --- | --- |
|  | **aOR (95% CI)** | **P-value** | **aOR (95% CI)** | **P-value** |
| Radiotherapy History | 3.42 (2.14 – 5.46) | <0.001 | 3.18 (1.92 – 5.25) | <0.001 |
| Age at Diagnosis | 1.07 (1.04 – 1.10) | <0.001 | 1.07 (1.04 – 1.10) | <0.001 |
| TSH Level | 2.01 (1.65 – 2.45) | <0.001 | 2.03 (1.66 – 2.48) | <0.001 |
| ER Status (Positive) | 2.47 (1.43 – 4.28) | 0.001 | 2.45 (1.41 – 4.25) | 0.001 |
| Family Hx of Thyroid CA | 3.05 (1.55 – 6.00) | 0.001 | 3.12 (1.58 – 6.15) | 0.001 |
| Molecular Subtype (TNBC)‡ | 0.25 (0.11 – 0.55) | <0.001 | 0.24 (0.10 – 0.54) | <0.001 |

Notes: aOR: adjusted odds ratio; CI: confidence interval. †The sensitivity analysis excluded 9 patients who were diagnosed with thyroid cancer within 2 years of completing breast cancer radiotherapy to rule out surveillance bias and non-causal associations. ‡Triple-negative breast cancer (TNBC) showed a protective effect (inverse association) compared to the Luminal A reference group.

**Supplementary Table S4.** Comparative performance of the XGBoost model in predicting Papillary Thyroid Microcarcinomas (PTMC) vs. Clinically Significant Thyroid Cancers

| **Performance Metric** | **PTMC Subgroup (≤ 1 cm)**  **(n = 92)** | **Clinically Significant Subgroup (> 1 cm)**  **(n = 108)** | **P-value†** |
| --- | --- | --- | --- |
| AUC-ROC (95% CI) | 0.862 (0.810 – 0.914) | 0.885 (0.835 – 0.935) | 0.482 |
| Sensitivity | 0.848 | 0.87 | 0.654 |
| Specificity‡ | 0.857 | 0.857 | - |
| Accuracy | 0.853 | 0.864 | 0.71 |
| Positive Predictive Value (Adjusted)§ | 0.144 | 0.148 | - |

Notes: † P-value for the comparison of AUC-ROC curves calculated using DeLong’s test. ‡ Specificity was calculated based on the shared control group (n=200) to maintain consistency. § Adjusted PPV was calculated assuming a 2% prevalence rate, consistent with the recalibration method described in Table 4. PTMC: Papillary Thyroid Microcarcinoma.
